# Expression profiling and analysis of some miRNAs in subcutaneous white adipose tissue during development of obesity

**DOI:** 10.1186/s12263-020-00666-0

**Published:** 2020-05-04

**Authors:** Elham M. Youssef, Asmaa M. Elfiky, BanglySoliman, Nourhan Abu-Shahba, Mahmoud M. Elhefnawi

**Affiliations:** 1 Biochemistry Department, National Research Centre, Cairo, Egypt; 2 Environmental and Occupational Medicine Department, Environmental Research Division, National Research Centre, Cairo, Egypt; 3 Department of Biochemistry, Faculty of Science, Ain Shams University, Cairo, Egypt; 4 Stem Cell Research Group, Centre of Excellence for Advanced Sciences, Department of Medical Molecular Genetics, National Research Centre, Cairo, Egypt; 5 Informatics and Systems Department, Engineering Research Division, National Research Centre, Cairo, Egypt; 6 Biomedical Informatics and Chemoinformatics Group, Center of Excellence for Advanced Sciences, Informatics and Systems Department, National Research Centre, Cairo, Egypt

**Keywords:** High-fat diet, Normal diet, microRNA, Expression profiling, Inguinal subcutaneous white adipose tissue

## Abstract

**Background:**

MicroRNAs are emerging as new mediators in the regulation of adipocyte physiology and have been approved to play a role in obesity. Despite several studies have focused on microRNA expression profiles and functions in different metabolic tissues, little is known about their response to nutritional interventions in white adipose tissue during obesity stages, and whether they differ in this response to weight-reduction strategy is poorly understood. Our objectives were to study the dysregulation of some miRNAs in subcutaneous inguinal white adipose tissue during weight change, expansion/reduction; in response to both a high-fat diet and switching to a normal diet feeding, and to evaluate them as potential biomarkers and therapeutic targets for early obesity management

**Method:**

A hundred 6-week-old male Wister rats were randomly divided into a normal diet group (N.D), a high-fat diet group (H.F.D), and a switched to a normal diet group (H.F.D/N.D). At the beginning and at intervals 2 weeks, serum lipid, hormone levels, total body fat mass, and inguinal subcutaneous white adipose tissue mass (WAT) measurements were recorded using dual-energy X-ray absorptiometry (DEXA). The expression levels of microRNAs were evaluated using real-time PCR.

**Results:**

Significant alterations were observed in serum glucose, lipid profile, and adipokine hormones during the early stages of obesity development. Alteration in rno-mir 30a-5p, rno-mir 133a-5p, and rno-mir 107-5p expression levels were observed at more than one time point. While rno-let-7a-5p, rno-mir 193a-5p, and rno-mir125a-5p were downregulated and rno-mir130a-5p was upregulated at all time points within 2 to 4 weeks in response to H.F.D feeding for 10 weeks. The impact of switching to normal diet has a reversed effect on lipid profile, adipokine hormone levels, and some miRNAs. The bioinformatics results have identified a novel and important pathway related to inflammatory signalling.

**Conclusion:**

Our research demonstrated significant alterations in some adipocyte-expressed miRNAs after a short time of high caloric diet consumption. This provides further evidence of the significant role of nutrition as an epigenetic factor in regulation of lipid and glucose metabolism genes by modulating of related key miRNAs. Therefore, we suggest that miRNAs could be used as biomarkers for adiposity during diet-induced obesity. Perhaps limitation in calories intake is a way to manipulate obesity and associated metabolic disorders. Further studies are needed to fully elucidate the role of microRNAs in the development of obesity

## Introduction

Obesity is a metabolic disorder increasing in prevalence worldwide and is a result of complex interactions of multiple factors including genetics and environment. Such disturbance which is associated with a higher incidence of many diseases, including diabetes, cardiovascular disease, and cancer, creates a global epidemic of major health concerns [1, 2]. It is now firmly established that diet is among the environmental factors that lead to metabolic alterations. Consuming high-calorie density food leads to increase fat accumulation in adipose tissue and consequently increases the levels of circulating free fatty acids in the blood. This leads to macrophage activation and the production of pro-inflammatory cytokines via Toll-like receptors, resulting in inflammation in adipose tissue [3, 4]. In experimental studies, when the mice have been received a high-fat diet (H.F.D), they develop insulin resistance and obesity in a manner similar to the progression of the disease in humans [5, 6]. Increased energy expenditure and decreased energy consumption are the most recommended lifestyle changes to reduce obesity and increase insulin sensitivity in the treatment of dietary obesity (DIO) and obesity-related disease. Generally, limitation in calories intake and fat diet percentage decreases body weight and improves insulin sensitivity. Additionally, the bodyweight reduction and improvement of insulin sensitivity could also be attained by reduction of the percentage fat diet, which is by switching from a HFD to a low-fat diet (LFD) [7].

MicroRNAs (miRNAs) are endogenous small RNAs that post-transcriptionally regulate gene expression. They have been shown to have important roles in many disease processes.

There is increasing evidence that miRNA plays an important role in the regulation of adipose tissue pathways that control a range of processes, including adipogenesis, insulin resistance, and inflammation [8, 9]. Recent studies have uncovered several miRNAs expressed in metabolic organs that could be used as possible therapeutic targets for obesity and its consequences. Among these microRNAs are Let 7, the potent regulator of glucose metabolism and peripheral insulin resistance [10], which showed significant alteration in obesity and metabolic disorders [11]. In previous studies, it manifests its effects by targeting high-mobility group AT-hook 2 (HMGA2), a transcription factor that regulates growth and proliferation in other contexts. These phenomena occur in part through the let-7-mediated repression of multiple components of the insulin-PI3K-mTOR pathway, including IGF1R, INSR, and IRS2. In addition, the mTOR inhibitor, rapamycin, abrogates Lin28a-mediated insulin sensitivity and enhanced glucose uptake [12].

Moreover, it was postulated that miR-103/107 are involved in regulation of insulin through caveolin-1. miR-107 contributes in inflammation and obesity in a Toll-like receptor 4/myeloid differentiation primary response 88 manner (TLR4/MYD88) in the activated macrophages located in the adipose tissue; it also is known to be a lipid-modulated miRNA [13, 14]. Another miRNA was also found to be involved in diet-induced obesity, miR-107. It is an intronic miRNA and is co-regulated with its respective host gene pantothenate kinase 1 (PANK 1) during 3T3-L1 adipogenesis [15]. Its expression has been found to be downregulated in a mouse model of genetic insulin resistance and obesity, possibly through a stimulated inflammatory pathway [15, 16]. Additionally, other studies have confirmed the significant downregulation of mir-107 in white adipose tissue (WAT) of obese mice in a TLR4/MYD88 manner [13, 17–22]. These findings revealed mir-107 link to adaptive and innate immune activation/polarization with the potential role in obesity-mediated inflammation [13] with respect to evidence that were reported by Daimiel-Ruiz L et al. in 2015 [23].

Additionally, mir-30 and mir-125a trigger molecular mechanisms associated with insulin resistance (IR) and inflammation in adipose tissue. miR-130 is induced by the inflammatory condition and upregulated causing adipocyte dysfunction. The mechanisms are targeting peroxisome proliferator-activated receptor γ (PPARγ) which is downregulated during the pro-inflammatory response and adipocyte dysfunction [24–26]. In human adipocytes, miR-193b controls adiponectin production via pathways involving nuclear transcription factor Yα and possibly nuclear receptor interacting protein 1 [27].

Currently, how changes in miRNA profiles might affect adipose tissue at the functional and molecular level and to what extent they differ in response to weight-control strategies are not well understood. This information is important to help control the development of dietary anti-obesity interventions for obese individuals.

This present study aimed to investigate miRNA dysregulation in adipose tissue during weight change stages, expansion/reduction, detect whether this dysregulation can be restored, and detect potential biomarkers and therapeutic targets for obesity. Also, determine the target genes and the significantly regulated pathways of each miRNA and consequently, enrich their functional role in obesity onset and progression.

## Material and methods

### Experimental animals

Diet-induced obese rat models were used in this study. A hundred 6-week-old Wister male rats were selected from the breeding colonies maintained in the Modern Veterinary House /Egypt. All rats were group-housed in clean cages labeled with time points, 6–8 rats/cage, and were placed in a temperature-controlled room with a 12:12-h light-dark cycle. They were classified into three groups according to the feeding protocol:

1. Group A (H.F.D). Rats in this group received a high-fat diet (H.F.D) for 10 weeks.
2. Group B (H.F.D/N.D). High-fat diet feeding followed by normal diet which means a group from the fattened rats in group a received normal diet for 8 weeks.
3. Control group in which rats received normal diet for 18 weeks (N.D).

Retro-orbital sinus blood samples were collected from five rats in each group (*n* = 5) at 0, 2, 4, 6, 8, and 10 weeks of H.F.D group and at 4 and 8 weeks for the (H.F.D/N.D) along with their control groups. The diets provided at least 3.85 kcal/g of energy [28]. The basal experimental diet was formulated to cover the nutrient requirements of the rats. The normal diet used in this experiment contained enough nutrient and energy to meet requirements for rats’ growth and development. Additionally, a high-fat diet was performed by adjusting the proportion of fat of normal diet based on previous studies [29, 30]. Fat content was 20% sheep tallow as presented in Table 1.

**Table 1:** Component standard animal diet (chow diet) and high-fat diet

Composition of the diets (g/kg diets)	Normal diet (chow diet)	High-fat diet
Carbohydrates (corn starch)	65%	50%
Proteins (casein)	23%	23%
Fats (corn oil or beef tallow)	6% (corn oil)	20% (sheep tallow)
Fibers (cellulose )	3%	3%
Vitamins/minerals mixture	1–4%	1–4%
Total	100%	100%

Then those rats were sacrificed for Dual-energy X-ray absorptiometry DEXA and inguinal subcutaneous tissue collection by dissecting it free of surrounding tissue and then frozen in liquid nitrogen from. Serum and fat tissue samples were stored at −70°C before being used for extraction of RNA or biochemical and hormone assays.

Stainless steel nipples for drinking and feeders allowing recording individual feed intake for each rat were supplied for each cage. Rats of all groups were kept under the same managerial conditions.

### Adipose tissue analysis

Weight changes and fat composition of inguinal subcutaneous adipose tissue using dual-energy X-ray absorptiometry (DEXA) were recorded at 0, 2, 4, 6, 8, 10 weeks and at 4, 8 weeks for the (H.F.D/N.D) along with their control rat groups. Histological and morphometric analysis were carried out in adipose tissue samples of all groups. Representative microscopic pictures of hematoxylin-eosin-stained sections were used [31].

### Biochemical analysis

Alteration in hormone levels during weight changes were detected by enzyme-linked immunosorbent assay (ELISA), while blood glucose level was measured using hexokinase colorimetric method. The other biochemical analysis, total cholesterol, triglycerides, and high-density lipoprotein (HDL) was determined by enzymatic methods (Spectrum kit, Egyptian Company for biotechnology (S.A.E,) Obour City, Cairo, Egypt). The low-density lipoprotein cholesterol was determined using the equation: LDL cholesterol = total cholesterol − HDL cholesterol − (Triglycerides / 5)

### RNA extraction

Total RNA from (100 mg) adipose tissues was isolated using miRNeasy Mini Kit (QIAGEN, GmbH, Hilden), according to the manufacturer’s instructions. RNA quantitation was done using NanoDrop Lite (ThermoScientific, Wilmington, DE).

### Quantitative RT-PCR

Differential expression of (miR-130a-5p, miR-30a-5p, miR-133-5p, let-7a-5p, miR-107-5p, mir-125a-5p, and mir-195-5p) were evaluated in inguinal subcutaneous white adipose tissue driven from HFD , HFD/ND, and ND groups using quantitative RT-PCR (qRT-PCR). The total purified RNA (1 μg) was reverse transcribed into cDNA using miScript II RT Kit (QIAGEN, GmbH, Hilden) according to the manufacturer’s protocol. miRNA expression was performed by real-time PCR method. The reaction mixture was carried out in a total volume of 20 μL containing 2.0 μL of cDNA (100 ng/μL), 10 μL of 2× SYBR Green PCR Master Mix, 2.0 μL of 10× miScript Universal Primer, 2.0 μL of 10× miScript Primer Assay specific for each mature miRNA, and the total reaction volume was completed up to 20 μL using nuclease-free water. PCR amplification was carried out as follows: 95 °C for10 min; 40 cycles in three steps: 94 °C for 15 se, 55 °C for 30 s and 70 °C for 30 s, followed by 4 °C forever. Validation of miRNAs was done on Rotor-Gene, Real-Time System (BIO-RAD, Hercules, CA). Experiments were run in triplicates. Relative expression levels of miRNAs were analyzed using the 2^ (−ΔΔCt) method using RNU6B expression level as a housekeeping gene.

### Statistics and data presentation

All data are presented as mean ± SEM. Comparisons between time-points (week 0, week2, week 4, week6, week8, and week10) were performed using the paired *t* test and non-parametric test. *P* < 0.05 was considered significant.

### Bioinformatics analysis

In silico analysis for the selected seven miRNAs which are well known to exhibit differential alterations in case of obesity onset. This was performed to explain our experimental results of the miRNAs expression and importantly, to reveal their mechanistic roles in obesity. The flow of the analysis based on finding out the target genes regulated by each miRNA and identifying the possible cellular pathways in which these target genes are involved.

### MicroRNA in silico target analysis

MicroRNA target analysis was done using miRWalk 2.0 server [http://mirwalk.uni-hd.de/] which is a database that gives both predicted and experimentally validated miRNA-targets [32]. The predicted and the validated target genes of each miRNA were obtained with cut off *P* value 0.05. Both predicted and validated miRNA targets were combined together for further undergo functional enrichment analysis.

### Functional enrichment analysis

Functional enrichment analysis for the miRNA target genes was done using the DAVID server [Database for Annotation, Visualization and Integrated Discovery], (https://david.ncifcrf.gov) [33, 34] in order to reveal the pathways of obesity where the predetermined miRNA targets are regulated. The most significantly enriched pathways were retrieved from Kyoto Encyclopedia of Genes and Genomes (KEGG) [35] and they were detected with *P* value threshold 0.05.

## Results

Our results cover two experimental periods extended for 18 weeks. The first feeding condition is a 10-week H.F.D feeding period while the second one is an 8-week switching to N.D feeding period.

### In response to high-fat diet feeding

**Fig. 1:**
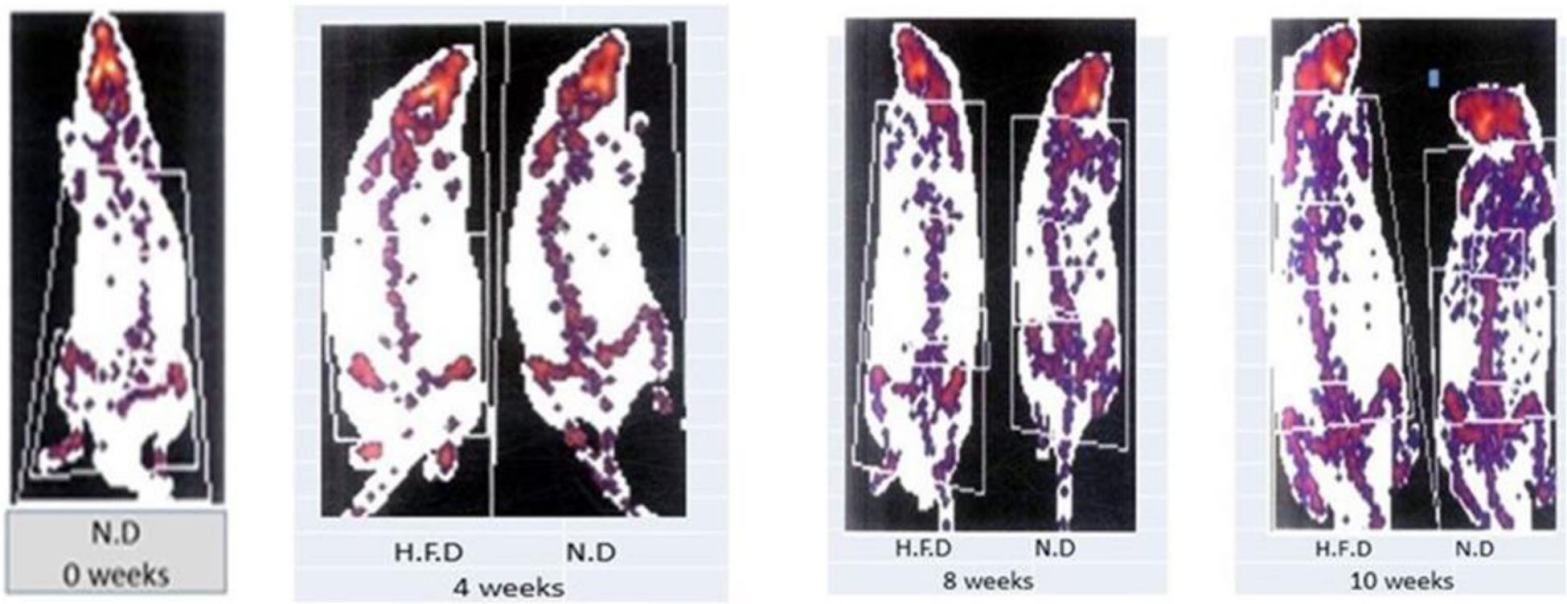
DEXA images represented total body fat and inguinal subcutaneous white adipose tissue fat during H.F.D and N.D groups

In our study, marked abnormalities were observed in high-fat diet (H.F.D) group rats during the early stages of fat diet feeding. Among these abnormalities are disturbance in adipokine, glucose, insulin levels, and pathophysiological changes. Moreover, the time course study of some miRNAs showed expressional changes in adipose tissue in different durations. The data presented in Table 2 show a significant difference in body weight of the high-fat diet group compared to the bodyweight of those fed on normal diet in 14 days after feeding. A gradual increase of body weight was observed with the highest significant (***P*** < 0.01) increase at week 10; however, a constant change in weight gain in the last feeding period was observed. It appeared reasonable to notice a gradual increase in total body fat mass and inguinal subcutaneous white adipose tissue mass in association with the gradual rise in body weight. The greatest fat mass increment was at weeks 8 and 10 (Table 2, Fig. 1).

**Table 2:** Changes of total body weight, total fat mass, and inguinal subcutaneous white adipose tissue mass in N.D and H.F.D groups

	0 week	2 weeks	4 weeks	6 weeks	8 weeks	10 weeks
Total body weigh t(g)						
N.D	110 ± 4.47	124± 6.2	157 ± 4.8	160±4.3	185±9.6	240 ± 2.8
H.F.D	115 ± 6.1	150 ± 5.0	170 ± 9.5	175 ± 12	215±8.2*	270 ± 6.1*
Total body weight change(g)	5	26	13	15	30	30
Total fat mass (g)						
N.D	0.9 ± 0.1	1.4 ± 0.06	2.8 ± 0.15	4.16 ± 0.4	23.3 ± 2.2	40 ± 2.8
H.F.D	1 ± 0.08	3.8 ± 0.6	5.3 ± 0.3	10.5 ± 1.0*	30 ± 2.9*	60.3 ± 1.2*
Total fat mass change (g)	0.1	2.44	2.52	6.3	7	20
Inguinal subcutaneous fat mass (g)						
N.D	0.09 ± 0.03	0.43 ± 0.1	0.72 ± 0.04	1.5 ± 0.2	2.96 ± 1.3	16.95 ± 0.2
H.F.D	0.08 ± 0.04	0.9 ± 0.2	1.5 ± 0.1	1.95 ± 0.26	5.7 ± 1.7*	22.9 ± 0.3*
Inguinal subcutaneous fat mass change(g)	0.01	0.47	0.7	0.9	1.59	5.98

Data represented as (mean ± SEM), (*n* = 5 per group).* *P* value, < 0.05 with paired *t* test

**Fig. 2:**
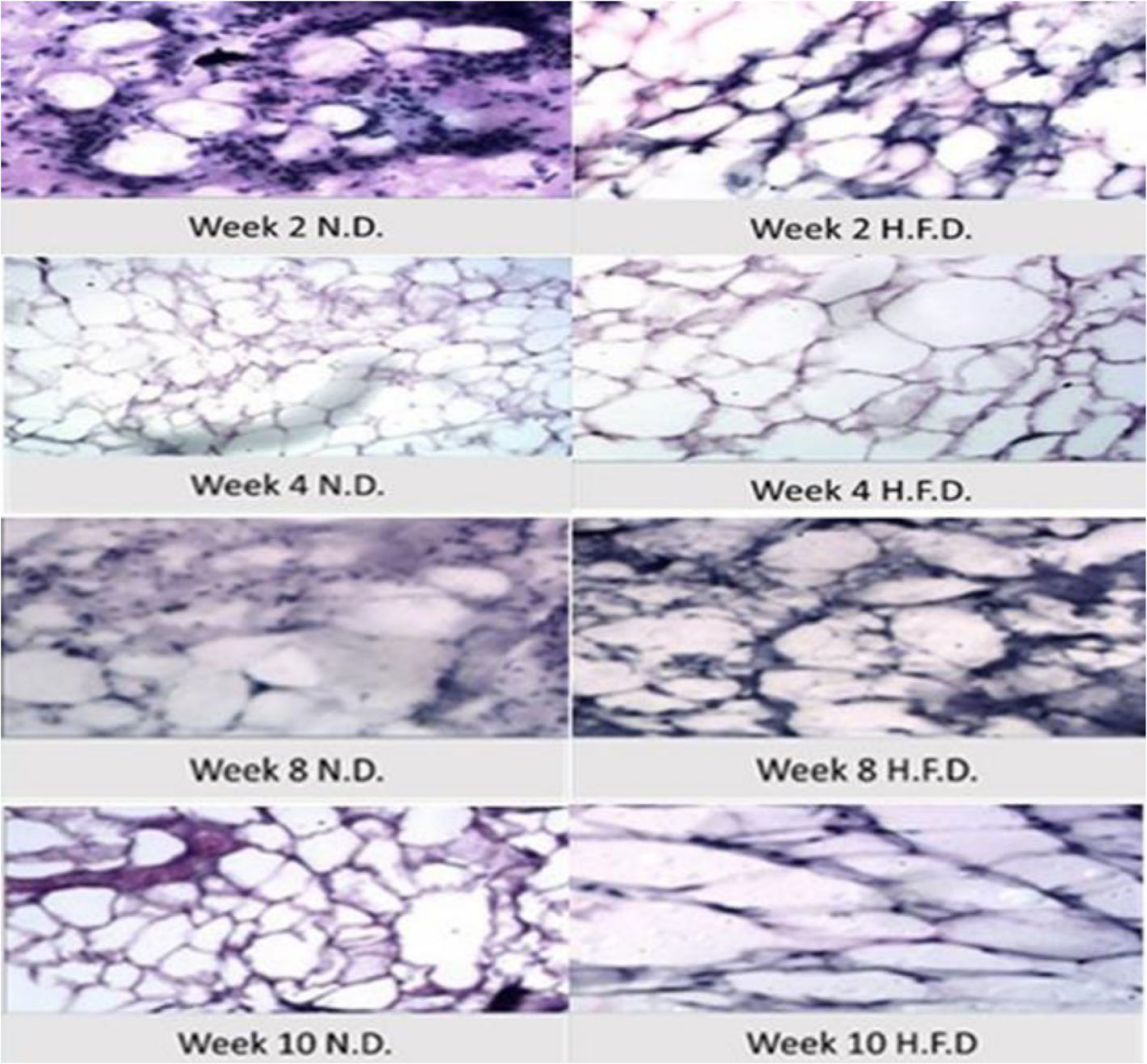
Adipose tissue morphology. Histology (hematoxylin and eosin-stained sections) of rat inguinal subcutaneous white adipose tissue showing difference in cell morphology between H.F.D and N.D groups at weeks 2, 4, 8, and 10

The effect of high-fat diet on fat cell morphology was observed under a microscope by H & E [36] (Fig. 2). The examined sections of subcutaneous inguinal white adipose tissue of rats fed on high-fat diet for 2 weeks revealed lobules of variable sized mature fat cells (average diameter 50 μm), separated by delicate fibrous septae. While those of normal group revealed lobules of variable sized mature fat cells (Average diameter 34.6 μm), with no detectable fibroplasia of inflammatory cellular infiltrate. Fat cell size increased until it reached the peak at week 8 and 10 as the lobules reached average diameter 225 μm separated by fibrous septae showing marked fibroplasia and mild inflammatory cellular infiltrate compared to control.

During the development of obesity in response to H.F.D, a clear change was observed mostly as a significant (***P*** < 0.05) increase in fasting glucose and insulin levels started at week 4 and week 6 respectively compared to normal diet group. Additionally, the blood lipid level in high-fat diet group markedly (***P*** < 0.05) increased reaching the peak at week 10. These changes included increased triglycerides, total cholesterol, and low-density lipoprotein and decreased high-density lipoprotein levels(Tables 3 and 4).

**Table 3:** Fasting glucose and insulin levels in N.D and H.F.D groups

	0 weeks	2 weeks	4 weeks	6 weeks	8 weeks	10 weeks
Blood glucose level (mg/dL)						
N.D	61 ± 8.9	75 ± 17.9	88 ± 19.2	86 ± 12.8	83 ± 14.9	92 ± 11.3
H.F.D	70 ± 7.9	93 ± 16*	128 ± 16*	112 ± 14*	115 ± 13*	130 ± 11.7*
Insulin level (uIu/mL)						
N.D	3.5 ± 0.5	3.35 ± 1.2	4.4 ± 0.5	4.45 ± 0.6	5.1±1.2	5.9 ± 0.6
H.F.D	3.2 ± 0.8	3.9 ± 0.7	4.8 ± 0.9	6.06 ± 1.04	9.3 ± 0.7*	9.95 ± 0.6*

Data represented as (mean ± SEM), (*n* = 5 per group).* *P* value, < 0.05 with paired *t* test

**Table 4:** Lipid profile levels in N.D and H.F.D groups

	0 week	2 weeks	4 weeks	6 week	8 week	10 week
Total cholesterol (mg/dL)						
N.D	30 ± 2.2	34 ± 3.4	46 ± 3.2	59 ± 3.2	64 ± 1.9	73 ± 2.9
H.F.D	34 ± 2.7	44 ± 1.9*	73 ± 2.5*	82 ± 1.7*	93 ± 3.4*	99 ± 1.6*
Triglycerides (mg/dL)						
N.D	52 ± 4.1	57 ± 5.2	60 ± 1.0	68 ± 1.4	78 ± 2.4	82 ± 2.7
H.F.D	59 ± 2.7	60 ± 1.2	70 ± 1.2	89 ± 2.2	94 ± 1.1*	94 ± 2.9*
High-density lipoprotein (mg/dL)						
N.D	13 ± 1.0	16.5 ± 0.5	26 ± 1.7	30 ± 0.8	41 ± 2.5	52 ± 3.3
H.F.D	14 ± 1.6	21 ± 0.7	39 ± 2.5	31 ± 1.3	33.5 ± 1.6*	29 ± 2.0*
Low-density lipoprotein (mg/dL)						
N.D	8.8 ± 0.7	7.0 ± 1.7	7.5 ± 0.6	5.4 ± 0.4	5.0 ± 0.7	3.3 ± 0.6
H.F.D	12 ± 1.4	15.4 ± 0.4	25 ± 4.2*	41 ± 1.9*	50 ± 10.4*	55 ± 8.5*

Data represented as (mean ± SEM), (*n* = 5 per group).* *P* value, < 0.05 with paired *t* test

Leptin concentration showed a significant (*P* < 0.05) increase as early as 4 weeks after the high-fat feeding diet was initiated (Table 5). The difference between the two groups H.F.D and N.D then kept constant during the 4 weeks of the experiment. The same pattern of response of leptin was observed in weight gain. In contrast, adiponectin level decreased significantly (*P* < 0.05) at week 2 reaching the lowest value at week 8 compared to control (Table 5).

**Table 5:** Levels of adipokine hormones in N.D and H.F.D groups

	0 week	2 weeks	4 weeks	6 weeks	8 weeks	10 weeks
Leptin Ug/L						
N.D	1.9 ± 0.2	2.18 ± 0.34	2.6 ± 0.12	23 ± 2.7	28 ± 3.1	39 ± 1.6
H.F.D	2.1 ± 0.6	2.12 ± 0.2	9.3 ± 0.6*	42 ± 3.9*	57 ± 7.9*	67 ± 8.6*
Adiponectin Ug/L						
N.D	10 ± 0.6	15.5 ± 2.7	73.4 ± 4.9	118 ± 5.9	257 ± 9.9	256 ± 11.8
H.F.D	12.5 ± 0.8	17.6 ± 1.2	20.3 ± 2.5*	36 ± 9.5*	148 ± 20*	189 ± 8.1*

Data represented as (mean ±  SEM), (*n* = 5 per group).* *P* value, < 0.05 with paired *t* test

The present study conducted profiling of some miRNAs to investigate the change in their expression pattern in inguinal subcutaneous white adipose tissue during high-fat diet consumption and during switching to normal diet feeding.

**Fig. 3:**
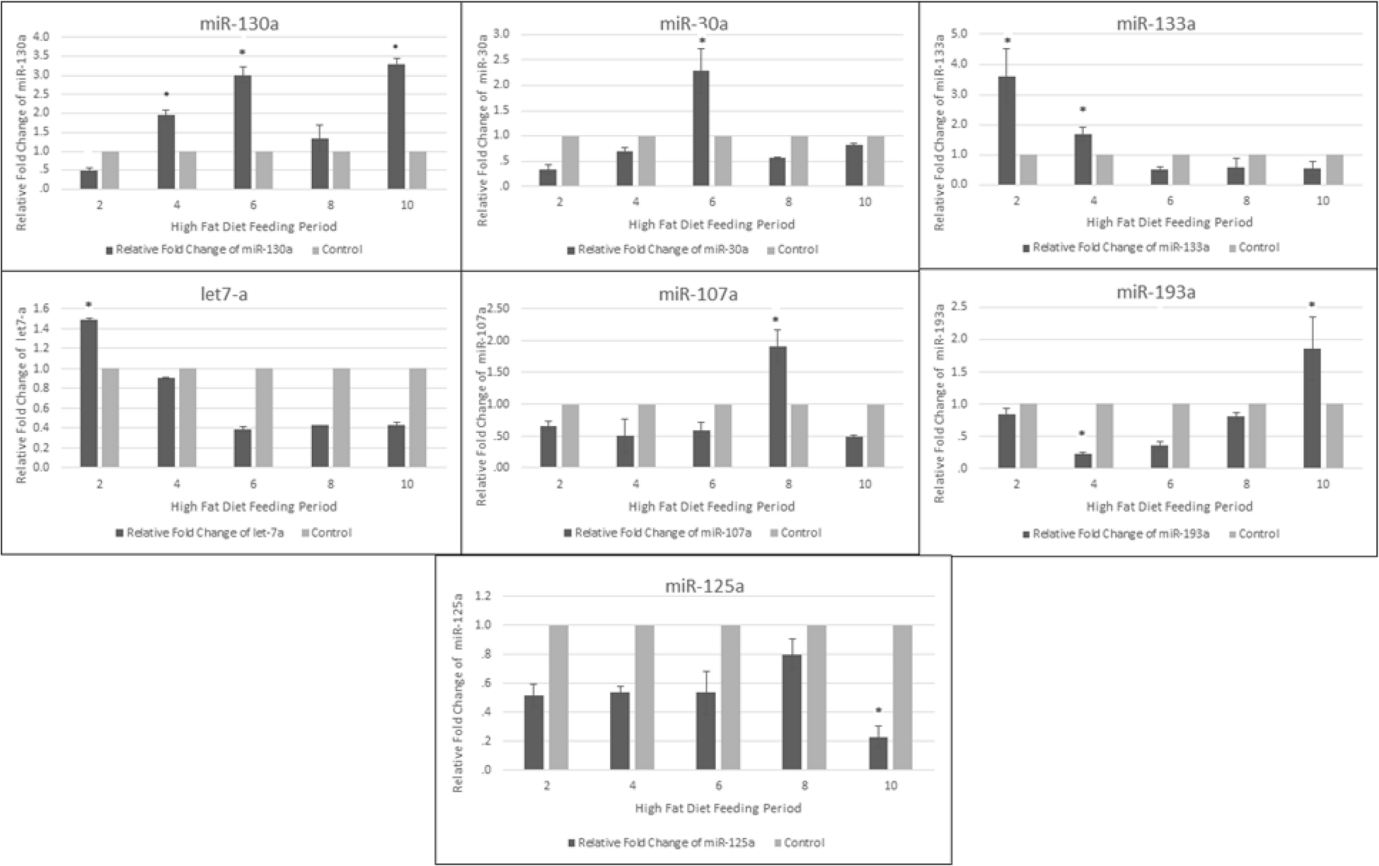
Relative fold change of mir 130a, 30a, 133a, let-7a 107,193a, and 125a in inguinal subcutaneous white adipose tissue during H.F.D group (*n* = 3 per group), * *P* value < 0.05 with paired *t* test

Our miRNA-profiling data analysis found that high-fat diet feeding exerted marked alterations on miRNA expression in such fat depot during the early stages of weight gain. The pattern expression of rno-mir-130a-5p in HFD group represented different alterations at more than one-time point. It showed a statistical gradual increase at week 4 and week 6 and reduced at week 8. However, at week 10 it statistically increased compared with control. Rno-mir-30a-5p of H.F.D group revealed a high statistical upregulation by 2.3-fold change at week 6 compared with control then reduced after 8 weeks compared with control. The expression level of rno-mir-133a-5p was significantly upregulated at weeks 2 and 4 then it was downregulated started from week 6 until the end of the experiment. Rno-let-7a-5p had nearly the same pattern of dysregulation of rno-mir-133a-5p. Moreover, rno-mir-107a-5p and rno-mir 193a-5p expression levels were significantly upregulated by 1.8-fold change at weeks 8 and 10 following fat diet feeding respectively compared with control. Regarding rno-mir 125a-5p, the dysregulation stared from week 2 until it reached a significant downregulation at week 10 compared with control (Fig. 3)

### In response to switching to normal diet feeding (H.F.D/N.D)

**Fig. 4:**
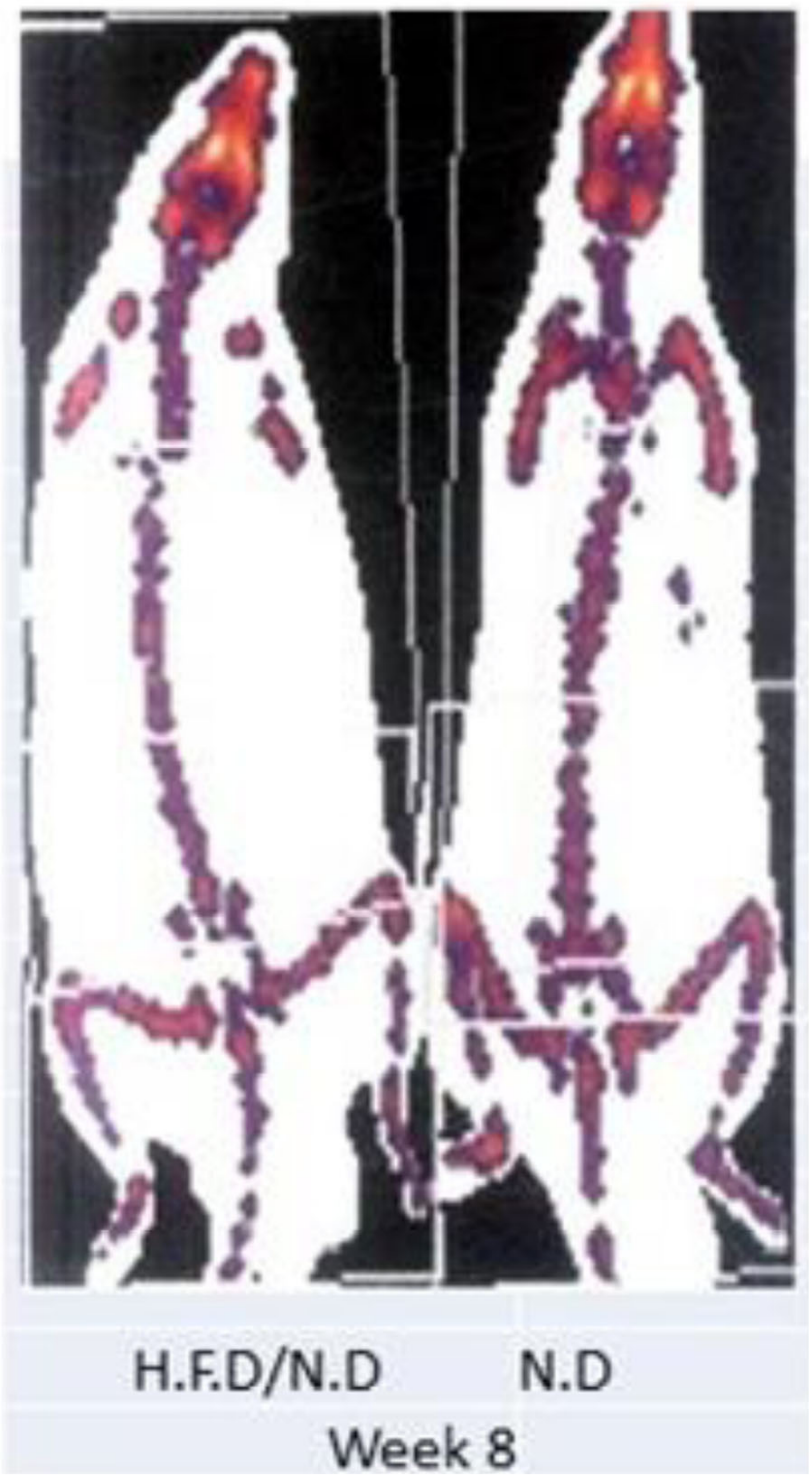
DEXA image represented total body and inguinal subcutaneous white adipose tissue fat of fattened rat 8 weeks after switching to normal diet feeding

A moderate change in weight and fat mass was observed as consequence of this feeding protocol comparing to control, therefore we were not able to detect significant changes of size and morphology of adipocytes after 8 weeks of returning rats to normal diet feeding and because weight changes were moderated (Table 6, Fig. 4), probably a kind of tiny local modifications could not be detectable using histological methods

**Table 6:** Changes of total body weight, total fat mass, and inguinal subcutaneous white adipose tissue mass in N.D and H.F.D/N.D groups

Duration/weeks	0 week	4 weeks	8 weeks
Total body weight (g)			
N.D	240 ± 2.8	255 ± 15	310 ± 29
H.F.D/N.D	270 ± 6.1*	265 ± 10	320 ± 24
Total body weight change (g)	30	10	10
Total fat mass (g)			
N.D	40 ± 2.8	42.8 ± 5.4	49.95 ± 4.5
H.F.D/N.D	60.35 ± 1.2*	44.04 ± 8	46 ± 9.9
Total fat mass change (g)	20.35	2.76	3
Inguinal subcutaneous fat mass(g)			
N.D	16.95 ± 0.2*	16.81 ± 1.2	19.09 ± 0.09
H.F.D/N.D	22.93 ± 0.3	18.08 ± 0.8	17.32 ± 1.5
Inguinal subcutaneous fat mass change (g)	5.98	1.27	2.77

Data represented as (mean ± SEM), (*n* = 5 per group).* *P* value, < 0.05 with paired *t* test

Switching from H.F.D to N.D was associated with a clear reversal in blood glucose and insulin levels at week 4 and 8. However, both levels were significantly higher compared with control at week 4 (Table 7).

**Table 7:** Fasting glucose and insulin levels in N.D and H.F.D/N.D groups

Duration/weeks	0 weeks	4 weeks	8 weeks
Blood glucose level (mg/dL)			
N.D	92 ± 11.3	100 ± 13.5	112 ± 16.7
H.F.D/N.D	130 ± 11.5*	123 ± 14*	118 ± 10
Insulin level (uIu/mL)			
N.D	5.5 ± 0.6	6.2 ± 1.1	7.4 ± 0.5
H.F.D/N.D	9.95 ± 0.06*	9.1 ± 2.0*	8.6 ± 1.3

Data represented as (mean ± SEM), (*n* = 5 per group).* *P* value, < 0.05 with paired *t* test

Other changes were also reversed significantly on switching to N.D like total cholesterol and high- and low-density lipoproteins. However, the alteration was moderate non-significant in triglycerides, leptin, and adiponectin levels (Tables 8 and 9).

**Table 8:** Lipid profile levels in N.D and H.F.D/N.D groups

Duration/weeks	0 week	4 weeks	8 weeks
Total cholesterol (mg/dL)			
N.D	73 ± 2.9	96 ± 6.1	103 ± 11
H.F.D/N.D	99 ± 1.6*	96 ± 8.6	89 ± 9.2*
Triglycerides (mg/dL)			
N.D	82 ± 2.7	86 ± 9.6	83 ± 9.8
H.F.D/N.D	94 ± 2.9*	81 ± 5.6	90 ± 6.5
High-density lipoprotein (mg/dL)			
N.D	52 ± 3.3	85 ± 5.5	65 ± 7.0
H.F.D/N.D	29 ± 2.0*	36 ± 3.9*	39 ± 3.1*
Low-density lipoprotein (mg/dL)			
N.D	3.3 ± 0.6	14.8 ± 1.9	19.9 ± 1.9
H.F.D/N.D	55 ± 8.5*	42 ± 6.1*	34.6 ± 4.2*

Data represented as (mean ± SEM), (*n* = 5 per group).* *P* value, < 0.05 with paired *t* test

**Table 9:** Levels of adipokine hormones in N.D and H.F.D/N.D groups

Duration/weeks	0 week	4 weeks	8 weeks
Leptin level (Ug/L)			
N.D	39 ± 1.5	36 ± 3	30 ± 4
H.F.D/N.D	67 ± 6.1*	39 ± 10	40 ± 24
Adiponectin level (Ug/L)			
N.D	256 ± 11.8	156 ± 16	52 ± 5
H.F.D/N.D	189 ± 14*	160±15	59 ±11.9

Data represented as (mean ± SEM), (*n* = 5 per group).* *P* value, < 0.05 with paired *t* test

**Fig. 5:**
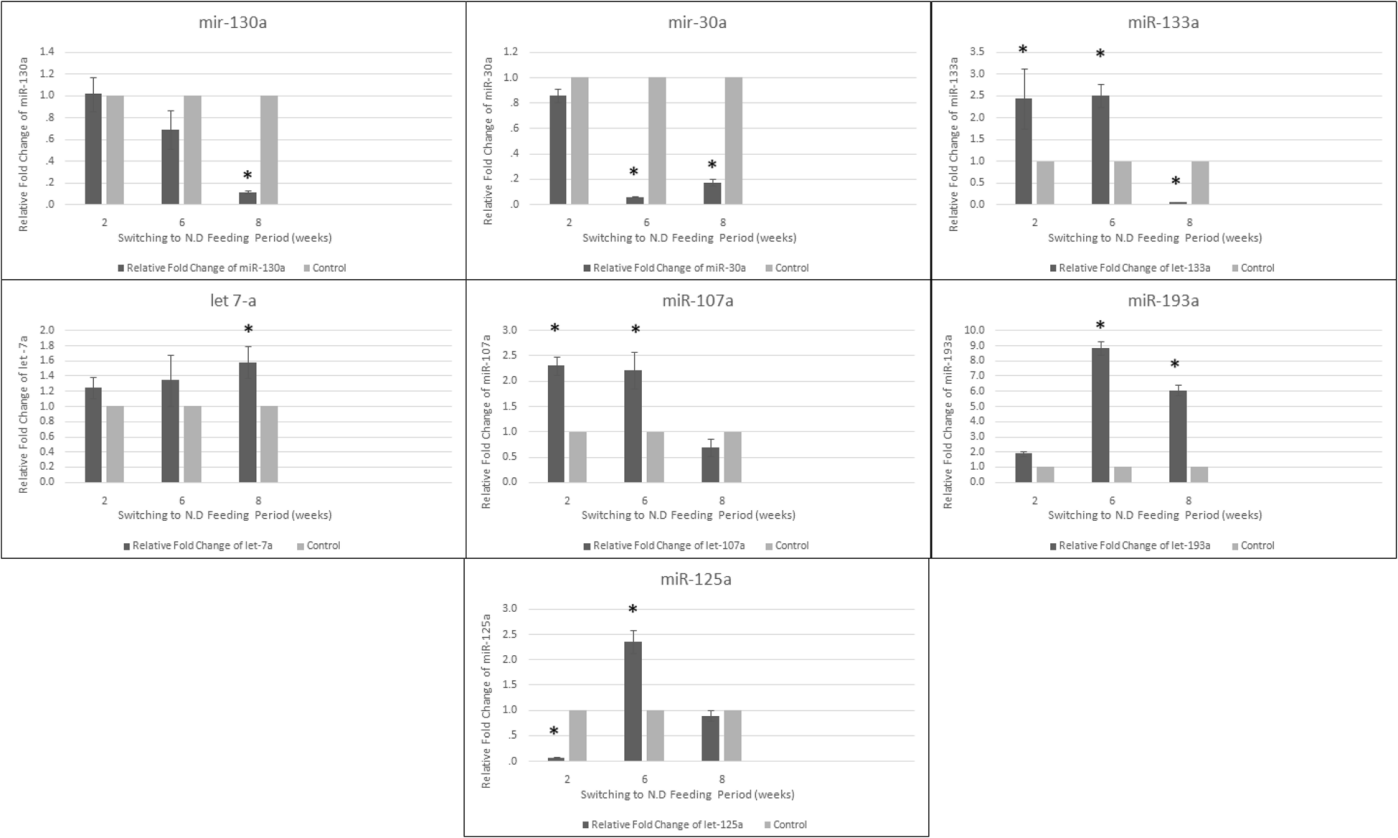
Relative fold change of mir-130a, 3a, 133a, let-7a, 107,193a, and 125a in inguinal subcutaneous white adipose tissue in response to switching to normal diet feeding (*n* = 3 per group), * *P* value < 0.05 with paired *t* test

Upon switching to N.D, 2 weeks were sufficient to lead to a significant upregulation of rno- mir-107a-5p, and rno-mir133a-5p, by 2.4-fold changes while rno-let-7a-5p showed late significant upregulation at week 8 in the subcutaneous inguinal white adipose tissue after the downregulations in response to H.F.D feeding. However, rno-mir 30a-5p was clearly maintained with a continuous significant downregulation at weeks 6 and 8. In contrast, rno-mir-193a-5p maintained with a continuous upregulation significantly at weeks 6 and 8. Additionally, rno-mir-125a-5p expression was significantly different along the time points (2, 6, and 8 weeks) compared with control (Fig. 5).

### Bioinformatics analysis results

The significantly predicted and validated target genes of each of our seven miRNAs in the context of obesity onset were identified using miRwalk target prediction tool. Then, the critical pathways of obesity, in which the pre-determined targets are implicated, were determined using DAVID functional enrichment tool. Interestingly, some of the determined pathways were considered novel as they have not been mentioned previously with the miRNA (Additional file 1: Table S1). miR-130-5p functional analysis has 26 significantly novel enriched pathways responsible for obesity development and progression. Interestingly, all of these pathways results supported miR-130-5p upregulation in response to H.F.D as results of upstream activator molecules enhancing miR-130-5p high expression. The most important pathways involved in this effect included endocytosis, focal adhesion, TGF-beta signaling pathway, insulin signaling pathway, and adipocytokine signaling pathway.

Regarding the results of functional analysis, miR-30a-5p has been found to regulate 31 pathways which involved in obesity. AMPK signaling pathway is the most significantly enriched pathway (*P* value 0.000000036). This pathway has been demonstrated to have a vital role for maintaining energy homeostasis of adipocytes and its activation protect against HFD-induced obesity by inhibiting adipogenesis, gluconeogenesis, and stimulates lipolysis, glucose consumption, and insulin sensitivity. Moreover, miR-30a-5p regulating its predicted target genes of AMPK signaling and consequently, miR-30a-5p may be a promising strategy for anti-obesity therapy.

Remarkably, endocytosis is the most significantly enriched pathway (*P* value 0.000000028).

mir-133a-5p has 34 significantly enriched pathways, and the novel pathways included fatty acid metabolism and regulation of autophagy. Remarkably, endocytosis is the most significantly enriched pathway (*P* value 0.000000028). This novel finding showing highly significant and coordinated regulation of miR-133a-5p of target genes of endocytosis mechanisms that reduce obesity. Overall, miR-133a-5p represents a potential drug target against obesity and type 2 diabetes through inhibiting adipogenesis and insulin resistance.

Let-7a-5p functional analysis has 32 significantly enriched pathways and the novel pathways included TNF signaling pathway and Notch signaling pathway which implied its involvement in obesity. Notably, MAPK signaling pathway and PI3K-Akt signaling pathway are the most highly statistically significant pathways (*P* value 0.000000014) which are regulated by let-7a-5p. Consequently, let-7a could inhibit all of these adipogenic properties of its targets and provide effective therapeutics against obesity.

mir-107-5p has 31 significantly enriched pathways and the novel pathways included Rap1 signaling pathway and Sphingolipid signaling pathway. Like mir-125, Rap1 signaling pathway is the most highly statistically significant pathway which is regulated by mir-107 but with higher number of predicted targets (187 target genes). In addition, Wnt signaling pathway is the second statistically significant pathway which evolved from our functional enrichment. mir-193a-5p functional analysis has 33 significantly enriched pathways and the novel pathways included Glucagon signaling pathway and glycolysis/gluconeogenesis. These pathways showed the enhanced anti-adipogenic role of mir-193-5p through regulating its target genes. In addition, MAPK signaling pathway represents the most significantly enriched pathway (*P* value 0.0000000011) which plays a critical role in adipogenesis and mir-193-5p can fine-tune the adverse events of this pathway to fight against obesity. Collectively, obesity-associated decrease in expression of miR-193a-5p may be responsible for its effects in adipose tissue.

Finally, mir-125a-5p functional enrichment analysis has shown 29 significant pathways and the novel pathways included cAMP signaling pathway and adipocytokine signaling pathway. Collectively, our findings showed the coordinated regulation of expression of miR-125a-5p and its target genes to improve obesity pathogenesis. Rap1 signaling pathway is the most highly statistically significant pathway evolved from our functional enrichment of miR-125a-5p.

## Discussion

During times of overfeeding, the size of adipocytes clearly increases due to the storage of extra triglycerides. Such expansion is strongly correlated with adipokine dysregulation, inflammation, insulin resistance, and other pathophysiological changes. These manifestations of high calories intake and fat accumulation involve a large number of cellular signaling, that itself is driven by various epigenetic modifications, most recently discovered, miRNA expression regulation [37–40]. Understanding these changes allows developing new therapeutics to change in the expression of miRNAs during obesity stages. We showed in our time course study expansion in inguinal subcutaneous white adipose tissue with a significant increase in fat mass determined by DEXA analysis when compared to control. The histological and morphometric study showed a clear increase in the size and the number of adipocytes at an early time point, week 4, during the high-fat diet feeding. The early increment in body weight in H.F.D. group was associated with the same pattern of response in leptin concentration which confirms the strong correlation between leptin and adiposity and its role in regulating appetite and body weight [41, 42]. Additionally, the disturbance in blood lipids, fasting glucose, and insulin levels in H.F.D group compared to N.D group suggested a rapid orientation of metabolism toward storage of lipid during the first weeks of overfeeding which is in line with previous studies [29, 43, 44]. The fat storage capacity in the progression of obesity [43, 45]. However, the late constant change difference in body weight appeared between our H.F.D and N.D groups suggested a sort of adaptation to high-fat feeding so as to control the gain in adiposity.

Remarkably, the fibrosis state in the later stages of the fat diet feeding period in our study empathized the role played by the fat diet in inflammatory events during the progress of obesity [46, 47]. More importantly, the remarkable feature of the process of adipogenesis is observed as an increased number of adipocytes in body fat depots. Previous work has focused on the regulation of this process through several adipocytes-selective microRNAs (miRNAs) and transcription factors that modulate adipocyte proliferation and differentiation. However, some miRNAs block expression of master regulators of adipogenesis [48–54].

In the context of these important regulatory roles of mRNAs, understanding in depth microRNA functions, their pathways, and target genes are possibly beneficial for weight control at an early stage to prevent health problems.

Therefore, the expression profiles of seven candidate miRNAs (rno-mir130a-5p, rno-mir30a-5p, rno-mir-133a-5p, rno-mirlet-7a-5p, rno-mir107a-5p, rno-mir193a-5p, and rno-mir125a-5p) were examined in our study. Few reports have been linking some of them with obesity, while the others were first identified.

As shown from the determined pathways resulted from the bioinformatics analysis, the seven miRNAs of interest regulate a variety of signaling pathways which are critical for obesity development and progression. Interestingly, some of these regulated pathways are novel and shared by most of the miRNAs in our study. Examples of the novel pathways include FoxO signaling pathway, Hippo signaling pathway, Rap1 signaling pathway, TNF signaling pathway, Toll-like receptor signaling pathway, chemokine signaling pathway, and adipocytokine signaling pathway. While examples of the common pathways included insulin signaling, fatty acid metabolism, PI3K/AKT signaling, MAPK signaling, and TGF-beta signaling.

The upregulation of miR-130a-5p in our study started from week 4 reaching the peak at week 10 suggested dysfunction in adipocytes from the early stages of high-fat dieting. Inflammation is a possible mechanism that may be responsible for this upregulation, i.e., high-fat diet-induced obesity recruits macrophages around the adipose tissue and promotes an inflammatory response [55]. miR-130 is then induced by this inflammatory condition and upregulated causing adipocyte dysfunction. The mechanisms are targeting peroxisome proliferator-activated receptor γ (PPARγ) which is downregulated during the pro-inflammatory response and adipocyte dysfunction. This was in line with Kim et al. [56] who observed that the levels of miR-130a and mir-130b were upregulated 3.9- and 2.9-fold change, respectively, through downregulation of its target gene, PPARγ in white adipose tissue from high-fat diet mice compared to control diet mice.

Experimental studies showed downregulation of miR-130b in obese or HFD mice [57, 58], while some other studies showed upregulation of miR-130b in ob/ob or HFD mice [58, 59]. These discrepancies among studies might be resulted from some other variables rather than the factors under investigation such as origin of fat cells (e.g., fat samples from subcutaneous or visceral fat depots, species, or strain of animals) and high-fat diet conditions (e.g., fat composition of diet, duration of HFD). Therefore, comprehensive and integrated analyses are required to explain the differential expression miRNAs in various obesity-related models.

In contrast, miR-30a-5p appeared to be downregulated during periods of HFD-feeding. However, switching back to normal diet did not change the expression pattern which is in a good agreement with Hsiehet al. (2015) [60]. Moreover, our gradual inflammation state of adipose tissue which started from delicate fibrous septae at week 6 of H.F.D feeding and reached a marked fibroplasia and mild inflammatory cellular infiltrate at weeks 8 and 10 supports the potential link of mir-30a-5p expression change with inflammation. The family of 30 miRNAs has been recently reported to modulate metabolic inflammation through Notch signaling genes, including Delta-like ligand-4 (DLL4), which are elevated in obesity and serve as a form of communication between macrophages and adipocytes. Also, Miranda Yang [61] conducted studies on adipose tissue macrophages (ATMs) using array analysis and PCR validation and revealed that miRNAs(-30a-5p, -30c-5p, and -30e-5p) were downregulated in obese ATMs suggesting that the miR-30 family plays an important role in macrophage phenotype. In our study, mir-30a-5p showed a sudden increase at week 6 which is not explained and needed further investigations. Interestingly, few reports revealed target sites for miR-30 on leptin gene, a gene encodes leptin hormone that is secreted by white adipocytes [62, 63]. Such hormone is well known to be linked to adiposity, appetite regulation, and insulin sensitivity; however, our abnormal leptin production by fat cell and the body response resistance in adiposity condition need further studies to validate miR-30a effect on leptin in such hormonal defect.

mir-133a-5p is known to be expressed in both brown and subcutaneous white adipose and directly targeting the 3′ UTR of PR domain containing 16 (Prdm16). It is the gradual lower expression that started from week 6 upon high-fat feeding in our study may reflect the differentiation of another kind of adipocyte which is called brown pre-adipocytes. This is in line with Liu W [64] who observed that the expression of miR-133a dramatically decreases in inguinal white adipose tissue along the commitment and differentiation of brown pre-adipocytes, accompanied by the upregulation of Prdm16. This adrenergic stimulus has been characterized as an important stimulator of brown adipocyte differentiation.

In previous work, miR-133a has been ranked among the differentially expressed miRNAs in association with a long-term high-fat diet and among the significantly downregulated miRNAs in white adipocytes [58, 65]. Based on these findings, mir-133a has been known to be important in obesity-related mechanism and inversely correlated with fasting glucose, HbA1c, and 2-h glucose tolerance [9]. These were consistent with our results which showed a significant decrease of miR-133a in WAT in HFD rat group and its significant increase on switch to normal diet. Notably, mir-133a was proposed as an important therapeutic target for the treatment of obesity because its overexpression has been found to decrease the expression of genes associated with general adipogenesis (*Pparg*, *Fabp4*, *Cebpb*, and *Adipoq*), beige adipogenic markers*(Tnfrsf9* and *Tmem26*), and brown adipogenic markers (*Prdm16*, *Pgc1a*, *Ucp1*, *Cidea*, and *miR*-*196a*).

In the present study, let-7-5p showed a decreased level of expression starting from week 4 and ended with a continuous decrease until week 10. Moreover, the significant increase of blood glucose and insulin levels at the same feeding period supports the hypothesis that miR let-7 might be the reason. Consequently, the gradual increase of body weight and fat mass reaching the peak in week 10 might result secondarily from disturbance of glucose level [9, 66].

Consistent with previous studies [67, 68], we have verified the downregulation of Let-7a-5p in the HFD rats compared to normal control and conversely, it has been found to be upregulated on switch to the normal diet.

Our current study validated a late upregulation of miR-107-5p in WAT at 8th week of the HFD period which would indicate a sort of adipose tissue inflammation. The downregulation observed in our study at week 10 may predict an adaptive inflammatory response [69].

The overall involvement of mir-107 in obesity has been revealed from the computational study which predicted that miR-107 affect multiple mRNA targets in pathways of energy and lipid metabolism in metabolic tissues including white adipose tissue [70].

The downregulation of miR-193 in WAT during and after HFD feeding-induced obesity has been previously reported [15, 19, 57, 58]. In addition, its downregulation caused a remarkable reduction in lipid accumulation and downregulation of adipogenesis markers, e.g., adiponectin [71]. In our work, the expression of this miRNA was consistently found to be downregulated in all HFD period except at week 10; this late significant increase of miR-193a-5p is in line with Ocłoń et al. [72] who conducted their study on a different kind of fat depot, epicardial adipose tissue, and concluded that miR-193 a is predicted to target phosphoenol pyruvate carboxykinase 2 (*PCK*)-2, *SOS*-*2*, tuberin (*TSC2*), or phosphatase and tensin homolog deleted on chromosome 10 (PTEN). These genes are involved in the insulin and adipocytokine signaling pathways. Taken together, supported clues about disturbances in glucose metabolism have been verified. In a NGS study, mir-193a was found to be among the obesity-associated miRs which significantly involved in oncogenesis [73]. The molecular mechanisms investigating the effects resulted from decreasing mir-193a in obese mice hasn't been clearly reported.

mir-125a-5p has been shown to be upregulated in mature adipocytes where it acts as a potent anti-adipogenic mir [74] through promoting adipocytes proliferation and inhibiting adipocytes differentiation. In addition, mir-125a has been reported to regulate fatty acid metabolism-related genes. Furthermore, miR-125a enhances insulin signaling and when it significantly downregulated it associated with insulin resistance in IR-3T3-L1 adipocytes [75].

Switching to a normal diet caused a non-significance reduction in leptin level, total body weight, and adiposity of the obese rat group and an increase in adiponectin level concentration. Notably, the upregulated miRNAs in HFD/ND were downregulated in obese rats and vice versa, implying that the downregulation of these miRNAs by obesity could be reversed by N.D treatment.

### Limitation

The study limitation included the H.F.D control group which is not continued in our experiment due to the small sample size. We considered that the results of 10th week (obesity rat) are a corresponding control of the weeks (2, 6, and 8). Also, this group achieved the purpose of significant weight-gain of obesity compared to control.

## Conclusion

In this study, we proved a clear dysregulation of miRNAs in important sites of energy storage, white adipose tissue, after short time of high caloric diet consumption. It also highlighted the improvement effect in dysregulated microRNA upon switching to normal diet. Additionally, it demonstrated the potential use of miRNAs as biomarkers for adiposity changes and as a therapeutic target in white adipose tissue during diet-induced obesity.

## Supplementary information

**Additional file 1: Table S1.** Enriched KEGG pathways for studied miRNAs.

## Data Availability

All data analyzed during this study are included in this article and its additional files.

## Abbreviations

N.D: Normal diet group
H.F.D: High-fat diet
WAT: White adipose tissue
DEXA: Dual-energy X-ray absorptiometry
DIO: Diet-induced obesity
miRNA: MicroRNA
